# Factors that determine cell fate in mitotically arrested cancer cells

**DOI:** 10.3389/fcell.2025.1691574

**Published:** 2026-01-12

**Authors:** Naghmana Ashraf, Roaa Kassim, Edward Goldstein, Taylor Landfair, Clarissa G. Nuñez, Jeffrey B. Arterburn, Charles B. Shuster

**Affiliations:** 1 Department of Biology, New Mexico State University, Las Cruces, NM, United States; 2 Department of Chemistry and Biochemistry, New Mexico State University, Las Cruces, NM, United States

**Keywords:** apoptosis, kinesin spindle protein, mitosis, mitotic slippage, phosphoinositide-3-kinase, Rap1

## Abstract

**Introduction:**

Cancer cells display a high degree of heterogeneity in their responses to mitotic arrest, from apoptosis during mitosis to surviving mitotic failure and continuing to progress through the cell cycle. Thus, understanding the basis for this variation may prove valuable for developing more effective chemotherapeutic strategies.

**Methods:**

A combination of biochemical and long-term live cell imaging approaches were applied to determine whether inhibition of Phosphoinositide 3-kinase (PI3K) signaling affected apoptosis in cancer cells arrested in prometaphase with a Kinesin Spindle Protein (KSP) inhibitor.

**Results:**

Dual inhibition of KSP and PI3K signaling induced apoptosis more effectively than mitotic arrest or PI3K pathway inhibition alone. Live cell imaging with probes for mitotic progression and apoptosis revealed that HeLa cells that died during mitotic slippage underwent apoptosis during prometaphase arrest, suggesting that PI3K inhibition dramatically shifted the dynamics of cell death. Similar potentiation of mitotic cell death could be detected in SiHa cells, whereas other cancer or non-transformed cell lines were not sensitized by PI3K inhibition. Expression of constitutively active Rap1, which modulates both cell adhesion and PI3K activity, significantly increased the duration of mitotic arrest in a PI3K-dependent manner. Moreover, activated Rap1 significantly increased the fraction of cells that slipped completely back into interphase prior to apoptotic cell death.

**Conclusions:**

These results shed insights into possible mechanisms by which cells may evade cell death during mitotic delay and suggest a strategy to optimize antimitotic interventions.

## Introduction

Inhibitors of spindle assembly represent a common strategy for cancer chemotherapy by inducing prolonged prometaphase delay and cell death (Jordan and Wilson, 2004; Salmela and Kallio, 2013). Suppression of microtubule dynamics or bipolar spindle assembly induces activation of the spindle assembly checkpoint (SAC), which maintains mitotic arrest by preventing cyclin degradation (Lara-Gonzalez et al., 2012). However, SAC suppression of the cyclin proteolysis is leaky, and over time, cyclin B is slowly degraded, leading to mitotic slippage and return to interphase without mitosis or cytokinesis (Brito and Rieder, 2006; Rieder and Maiato, 2004). In addition to mitotic slippage, leaky Anaphase Promoting Complex (APC)-mediated protein degradation can lead to loss of centrosome integrity and cohesion fatigue (Chiang et al., 2018; Karki et al., 2017), which can further compromise the fidelity of mitotic progression. However, the primary effect of prolonged mitotic arrest is cell death, and despite the use of antimitotic drugs in the clinic for decades, critical questions remain regarding the association between prolonged mitotic delay and apoptosis.

One underappreciated aspect of antimitotic strategies is that cells display a surprising heterogeneity of responses to prolonged mitotic delay. Cells might complete cell division with aneuploid daughter cells, die during mitosis or slip out of mitosis where cells might die, undergo G1 arrest, or continue to replicate and form tetraploid cells, and these heterogeneities exist between cell lines and between individual cells, both *in vitro* and *in vivo* (Gascoigne and Taylor, 2008; Orth et al., 2011; Orth et al., 2008; Shi et al., 2008). There are multiple potential mechanisms that may trigger apoptosis after prolonged mitotic arrest (Orth et al., 2012). Mitotic delay has been associated with increased DNA damage and partial caspase activation, as well as CDK1-mediated phosphorylation and degradation of the anti-apoptotic protein Mcl-1 (Harley et al., 2010). Other lines of evidence implicate the maintenance of energy levels, as evidenced by a loss in mitochondrial membrane potential before the onset of apoptosis in HL60 promyelocytic leukemia cells, which suffer mitotic death when treated with kinesin spindle protein (KSP) inhibitors (Tang et al., 2011). Thus, developing optimal antimitotic chemotherapeutic strategies is hindered by a lack of basic understanding of why cells die and why they respond differentially to mitotic delay.

The phosphoinositide-3-kinase (PI3K) signaling pathway is critical for cell proliferation and survival and is dysregulated in many cancers (Bachman et al., 2004; Karakas et al., 2006). When unregulated, increased AKT activity inhibits pro-apoptotic Bcl-2 proteins, thus suppressing apoptosis during tumor development and contributing to drug resistance (Franke et al., 2003; West et al., 2002). Thus, the PI3K/AKT/mammalian target of rapamycin (mTOR) pathway represents a powerful target for therapeutic intervention (Glaviano et al., 2023). However, despite its well-documented roles, PI3K inhibition alone does not significantly culminate in apoptosis in some cell types (Hou et al., 2012; Okkenhaug et al., 2016; Peng et al., 2022). Although PI3K inhibition alone may not be effective in chemotherapy, combining it with antimitotic agents may show promising results for cancer treatment when compared to traditional antimitotic strategies (Hu et al., 2002; Wallin et al., 2012). In this report, we explored a combination of antimitotic agents that target both KSP and the PI3K pathway by applying biochemical, morphological, and live-cell imaging approaches to examine the behavior of HeLa cells, which are reported to undergo apoptosis after undergoing mitotic slippage (Rodriguez et al., 2011). Live-cell imaging of HeLa cells revealed that the kinetics of cell death were significantly accelerated in the presence of inhibitors of the PI3K pathway during mitotic arrest. This effect was also observed in SiHa cells, which normally survive mitotic arrest. Our work also showed that activated Rap1, which dramatically flattens cells during mitosis, significantly increased the duration of mitotic arrest in a PI3K-dependent manner. Together, these data suggest that the ability of cells to survive prolonged mitotic arrest may partially be a function of PI3K signaling and also provide a possible avenue for combinatorial therapy, in which inhibition of pathways regulating cell survival could increase the efficacy of KSPIs in the clinical setting.

## Materials and methods

### Cell culture and transient transfections

All cell lines were obtained from the American Type Culture Collection (ATCC). HeLa (RRID: CVCL_0030), SiHa (RRID: CVCL_0032), and C33A (RRID: CVCL_1094) cells were cultured in Eagle’s Minimum Essential Medium (EMEM) supplemented with 10% fetal bovine serum (FBS) (Atlanta Biological), 2.5 mM L-glutamine, 1.0 mM sodium pyruvate, and 2.5 mM sodium bicarbonate (Lonza). Ca Ski (RRID: CVCL_1100) cells were cultured in RPMI-1640 medium (ATTC) supplemented with 10% FBS (Atlanta), 2 mM L-glutamine (Sigma, MA), 1.0 mM sodium pyruvate, and 2.5 mM sodium bicarbonate (Lonza). U-2 OS (RRID: CVCL_0042) and telomerase-immortalized human retinal pigmented epithelial cells (RRID: CVCL_4388) were cultured in DMEM: F-12 medium (ATCC) supplemented with 10% FBS (Atlanta Biological), 5 mM sodium bicarbonate (Lonza), and 1.0 mM sodium pyruvate. All cells were grown in a humidified incubator at 37 °C and 5% CO_2_ and screened for *Mycoplasma* using a Universal *Mycoplasma* Detection Kit (ATCC, # 30-1012k).

Transient transfection of single plasmids or co-transfection with cyclin B-Venus (Addgene, RRID: Addgene_26062) was performed using Lipofectamine 2000 (Thermo Fisher, #11668019). After 6 h of transfection, cells were allowed to grow in fresh media overnight, followed by drug treatment and imaging.

### Drug treatments

Cells were arrested in mitosis with methoxytrityl-S-cysteamine (MSTNH2), at 500 nM, a concentration that resulted in inhibition of spindle bipolarity in 100% of mitotic HeLa cells and was ≥ EC50 for all cell types in the NCI60 panel (Rodriguez et al., 2011). To test for potentiation of cell death during mitotic arrest, cells were cultured in MSTNH2 in the presence or absence of the following drugs at concentrations based on the following studies: 20 μM LY294002 (Selleckchem, #S1105) (Li et al., 2014), 20 μM AZD5363 (Selleckchem, #S8019) (Zhang et al., 2016), 500 nM rapamycin (Selleckchem, #S1039) (Yellen et al., 2011) and 10 nM defactinib (Selleckchem, #S7654) (Le Large et al., 2021). Cells were treated with 1 μM staurosporine as a positive control for apoptosis (Cell Signaling Technology, #9953).

### Immunofluorescence

HeLa cells were seeded on coverslips and fixed and processed for immunofluorescence, as previously described (Karki et al., 2017), using rabbit anti-cleaved caspase-3 (1:500, Cell Signaling, #9661) and mouse anti-tubulin (1:500, Sigma, #T5168). Primary antibodies were detected with Alexa Fluor 488 donkey anti-mouse IgG (RRID: AB_2866493) and Alexa Fluor 568 donkey anti-rabbit IgG (RRID: AB_2534017) (Thermo Fisher). DNA was visualized with 1 μg/mL Hoechst 33342 (Thermo Fisher, #62249). Images were acquired using a Zeiss Axiovert 200M inverted microscope equipped with epifluorescence optics and an Apotome structured illumination module. Images were exported, and figures were prepared using Photoshop CS (RRID: SCR_014199).

### Western blot analysis

HeLa cells were lysed with RIPA buffer (25 mM HCl, PH 7.6, 150 mM NaCl, 5 mM EDTA, 1% Triton X-100, 1% sodium deoxycholate, and 0.1% SDS) supplemented with 220 μM PMSF, 200 μM DTT, and protease inhibitor cocktail (VWR, #97063-010). Proteins were resolved on 4%–15% SDS-PAGE gels (Bio-Rad) and transferred to Immobilon-P (Millipore) membranes blocked in 5% milk diluted in 1x TBST (1 M Tris-HCl, 1.5 M NaCl, 0.5% Tween-20, pH 7.5) for 1 h at room temperature and then probed with rabbit anti-poly (ADP-ribose) polymerase (PARP) (1:1000, Cell Signaling, #9542) or mouse anti-β-actin (1:3000, Sigma-Aldrich, #A1978) at 4 °C overnight. Primary antibodies were detected with peroxidase-conjugated secondary antibodies (1:3000, GE Healthcare, #NA9340V, #NXA931) and incubated at room temperature for 45 min. Blots were developed using a Clarity Western ECL substrate kit (Bio-Rad) and imaged using a Molecular Imager ChemiDoc XRS System (Bio-Rad). For the Western blots shown in Supplementary Figure S2, cells were lysed in CST buffer (20 mM Tris-HCl, pH 7.5, 50 mM sodium fluoride, 1 mM EGTA, 1 mM EDTA, 0.1% SDS, 150 mM NaCl, 2.5 mM sodium pyrophosphate, 1 mM Na_3_VO_4_, 2 mM beta-glycerophosphate, 10 nM and Calyculin A) supplemented with protease inhibitors (Roche, cOmplete, #11836170001) at 4 °C for 15 min with shaking in lysis buffer. Lysates were centrifuged at 21,300 x g for 20 min at 4 °C. Protein levels were quantified using the Pierce BCA protein assay kit (Thermo Fisher, #23225). Blots were probed with rabbit anti-pAKT (1:1000, Cell Signaling, #9614S), rabbit anti-S6K (1:1000, Cell Signaling, #9202L), and rabbit anti-pS6 240 (1:1000, Cell Signaling, #2215S). Blots were developed using SuperSignal™ West Femto Maximum Sensitivity Substrate (Thermo Fisher, #34096) for 5 min and imaged using an Azure Biosystems 300. Eight-bit images were exported, and figures were prepared using Adobe Photoshop CS (RRID: SCR_014199). Cleaved PARP was quantified and normalized against actin using Fiji (RRID: SCR_002285), and statistical analysis and graphical representation of the data were performed using GraphPad Prism software (RRID: SCR_002798).

### Expression constructs and sub-cloning

A single-channel caspase reporter (Bhola and Simon, 2009) was constructed with a C-terminal peptide 2A site for bicistronic expression of a second open reading frame. Briefly, a Gblock containing an N-terminal myristoylation sequence, a caspase 3 cleavage site (DEVD), mCherry, and a C-terminal 2A sequence (Myr–DEVD–mCherry–2A) was synthesized (Integrated DNA technologies) and amplified by polymerase chain reaction (PCR) using primers containing an XhoI restriction site in the flanking sequence. The PCR product was cloned into XhoI-cut pCS2P+ (Addgene; RRID: Addgene_17095) using In-Fusion Cloning (Takara, #638912) and confirmed by Sanger sequencing. The resulting expression plasmid was designated as pMDM-2A.

For simultaneous labeling of microtubules along with caspase activation, the open reading frame of mCerulean3-tubulin (Addgene; RRID: Addgene_55450) was amplified with primers containing an EcoRV site, and the resulting PCR fragment was sub-cloned into EcoRV-digested pMDM-2A using In-Fusion Cloning (Takara, #638912), which was then confirmed by sequencing. The resulting expression construct was designated as pMDMCT. To generate a probe that simultaneously monitors mitochondrial outer membrane permeabilization (MOMP) and caspase activation, the mitochondrial import sequence of SMAC protein (Albeck et al., 2008) fused to the N-terminus of the mNeonGreen fluorescent protein (IMS-mNeon) was amplified by PCR and sub-cloned into the EcoRV site of pMDM-2A, and the resulting construct was designated as pMDM-IMS-mNeon. To investigate the role of cell adhesion, the open reading frame of Human Rap1A (HsRap1A) was amplified from a synthetic Gblock and sub-cloned into EcoRV-cut pMDM-2A. The subsequent expression plasmid was designated as MDM-2A-Rap1. Constitutively, active (Q63L) and dominant negative (S17N and S17A) mutants of MDM-2A-Rap1 were generated using the QuickChange II site-directed mutagenesis (Agilent, #200521) following the manufacturer’s specifications.

### Long-term time-lapse imaging

HeLa cells expressing fluorescent reporters were imaged using an Andor Dragonfly 505 spinning disk confocal system mounted on an Olympus IX83 inverted microscope. HeLa cells were plated on 35-mm glass-bottomed MatTek dishes (Mattek, #P35G-1.5-14-C) and transfected as described above. The following day, cells were treated with the drug for 4 h–8 h before being placed in an OKO-TOUCH pre-warmed (37 °C) temperature-controlled microscope stage supplied with 5% CO_2_. Z-stacks were acquired at 2-min intervals until the cells divided or underwent apoptosis by using a ×60 apochromatic silicon objective (NA 1.30) with a Tokai Hit objective warmer and an Andor iXon 888 EMCCD camera driven by Andor Fusion software. Cell fates of individual cells undergoing mitotic arrest and apoptosis were blinded for analysis and scored twice. Projection image sequences were created in Imaris (Oxford) and exported as 8-bit image stacks for movie and figure preparation in Fiji (RRID: SCR_002285) and Adobe Photoshop CS (RRID: SCR_014199).

High-throughput analysis of cellular responses to mitotic arrest was performed using an IncuCyte S3 live-cell analysis system (Essen Bioscience, Ann Arbor, MI). Cells plated in 24- or 96-well dishes were cultured in the presence of carrier control (DMSO) or small-molecule inhibitors and Annexin V Green or Caspase 3/7 Green Dye (Essen BioScience) to label apoptotic cells. Fluorescence and phase contrast imaging were performed at 3 or 9 locations per well (for 96- or 24-well plates, respectively) hourly for up to 48 h at 37 °C and 5% CO_2_. IncuCyte S3 software was used to program image acquisition and perform data analysis. To quantify cell fates of individual cells, video data from each condition for three experimental replicates were exported as TIF files, and the total time spent in mitosis, along with the fate of the cells, was measured manually.

### Statistical analysis

Statistical analysis was performed using one or two-way analysis of variance (ANOVA) test, followed by Tukey–Kramer *post hoc* test with a 95% confidence interval using GraphPad Prism 8 software. For all percentage data, data were arcsin-square root-transformed, followed by one-way ANOVA and Tukey–Kramer *post hoc* test. Significance in figures was denoted as *p < 0.05, **p ≤ 0.01, ***p ≤ 0.001, and ****p ≤ 0.0001.

## Results

### Simultaneous inhibition of spindle bipolarity and PI3K potentiates mitotic cell death in HeLa cells

**FIGURE 1:**
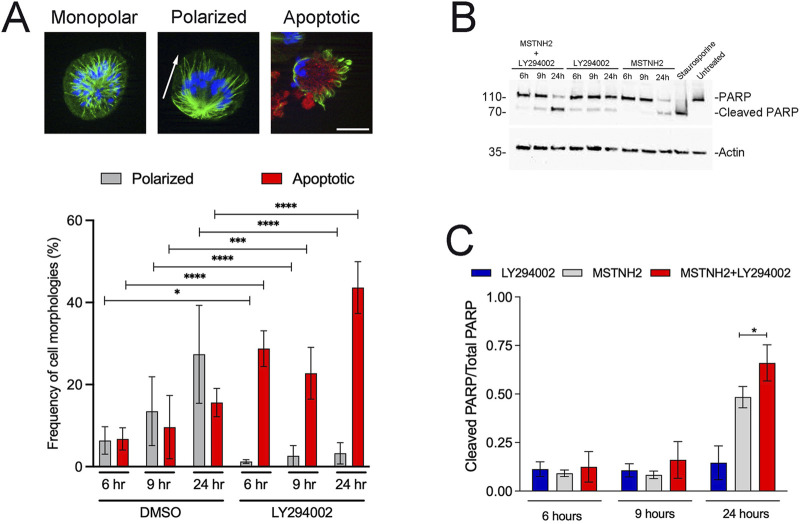
HeLa cell responses to prolonged mitotic delay. **(A)** HeLa cells were arrested in mitosis with 500 nM MSTNH2 for up to 24 h, and then fixed and probed for tubulin (green), activated caspase-3 (red), and DNA (blue) localization. Mitotic cells displayed three different morphologies: cells arrested in prometaphase with monopolar spindles (left); cells slipping out of mitosis with the monopolar spindle polarized in one direction (middle panel; axis of polarity denoted with an arrow); cells undergoing apoptosis, as identified by the presence of cleaved caspase-3 signal (right, red). Bar, 10 µm. The graph depicts the changing distribution of polarized and apoptotic morphologies over the course of 24 h, with ∼200 cells scored per biological replicate (three biological replicates per condition per experiment; six experimental replicates total). Error bars represent SD. **(B)** Cell lysates were generated from HeLa cells treated with 500 nM MSTNH2, 20 μM LY294002, MSTNH2+LY294002, or 1 μM staurosporine (positive control for PARP cleavage and apoptosis) and probed for PARP and actin. **(C)** Quantification of PARP conversion from its full-length (116 kd) to the cleaved 89-kd proteolyzed product; error bars represent SD of three independent experiments. *p < 0.05 and ****p < 0.0001.

Disruption of microtubule dynamics results in prometaphase arrest by the spindle assembly checkpoint (Lara-Gonzalez et al., 2012). Cells that cannot satisfy the checkpoint follow several possible cell fates, including apoptosis or slippage back to the interphase, where they may die, be arrested in G1, or attempt another round of division (Topham and Taylor, 2013). HeLa cells are reported to die shortly following mitotic slippage (Orth et al., 2008), and to further investigate the response of HeLa cells to mitotic delay, they were treated with the KSP inhibitor, MSTNH2 (Rodriguez et al., 2011), and examined for morphological and biochemical markers of mitotic arrest, slippage, and apoptosis (Figure 1). Similarly to other cells treated with KSP inhibitors, HeLa cells formed monopolar spindles with chromosomes arranged in a rosette pattern (Figure 1A, left panel). Cells undergoing mitotic slippage (due to leaky Anaphase Promoting Complex activity during mitotic arrest) experience a decrease in the CDK1 activity, resulting in a shift in microtubule dynamics, and the spindle undergoes random polarization in one direction (Figure 1A, middle panel). Cells undergoing apoptosis could be identified by the presence of cleaved caspase-3 in polarized cells (Figure 1A, right panel), and quantification of these morphologies revealed that over time, the number of monopolar cells decreased, whereas that of polarized and apoptotic cells increased (Figure 1A).

PI3K regulates a wide variety of downstream effectors that promote cell growth and survival (Glaviano et al., 2023), and activated variants of its downstream effector, AKT, protect cells from antimitotic drugs (Ma et al., 2019; VanderWeele et al., 2004). To determine if PI3K signaling determines cell fate in response to mitotic arrest, HeLa cells were treated with MSTNH2 alone or in combination with a PI3K inhibitor (LY294002) and probed for morphological or biochemical markers for mitotic arrest and apoptosis (Figures 1A–C). MSTNH2 treatment alone resulted in a gradual increase in the number of polarized and apoptotic cells, whereas the combined treatment resulted in significant increases in apoptotic cells (Figure 1A), both at early (9h) and late (24h) time points. Interestingly, there was a significant decrease in cells undergoing mitotic slippage (polarized cells) in the combination treatment (p = 0.028, 0.002, <0.0001 for 6, 9, and 24 h, respectively), suggesting that cells were dying before mitotic slippage. Western blotting revealed that in MSTNH2-treated cells, caspase-mediated cleavage of poly (ADP-ribose) polymerase (PARP) could be detected by 24 h (Figures 1B,C), but the combinatorial treatment was more effective at promoting PARP cleavage than mitotic arrest or PI3K inhibition alone (Figures 1B,C).

Protein kinase B or AKT lies downstream of PI3K, and to confirm that the inhibition of the PI3K pathway could potentiate cell death during mitotic delay, HeLa cells were subjected to mitotic arrest in the absence or presence of the AKT inhibitor AZD5363 and analyzed by immunofluorescence and Western blotting (Supplementary Figure S1). Differences in the fraction of apoptotic cells between the treatments (MSTNH2 vs. MSTNH2±AZD5363) could be detected in the earliest time points and were pronounced by 24 h (Supplementary Figure S1A). Similarly, Western blotting revealed that the combined treatment of MSTNH2/AZD5363 significantly increased caspase-3-mediated PARP cleavage compared to MSTNH2 alone (Supplementary Figure S1C). AZD5363 alone promoted some PARP cleavage (Supplementary Figure S1B), but this was not supported by the cell morphology assay (Supplementary Figure S1A), suggesting that AZD5363 induced partial caspase activation without triggering full apoptosis, similar to what has been observed in other studies (Orth et al., 2012).

### Inhibition of PI3K/AKT alters cell fate in response to mitotic delay

**FIGURE 2:**
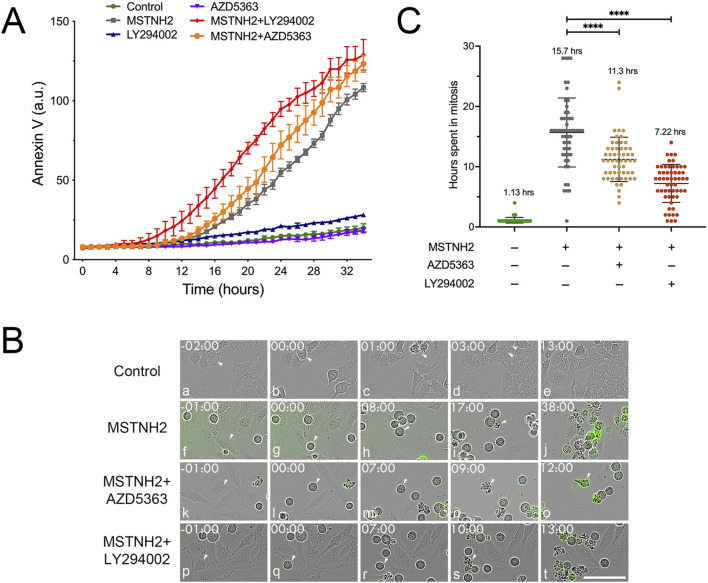
Time-resolved analysis of HeLa cell responses to mitotic delay in response to KSP and PI3K signaling inhibitors. **(A)** HeLa cells were cultured in the presence of green fluorescent annexin V and combinations of KSP inhibitor (MSTNH2) and PI3K pathway inhibitors (Akt, AZ5363; PI3K, and LY294002). Apoptosis and cell morphology were monitored hourly by throughput microscopy (four wells per condition and eight data points per well). Disruption of PI3K signaling significantly accelerated apoptosis in HeLa cells undergoing mitotic arrest as compared to cell death in controls, MSTNH2-treated, or PI3K inhibition alone. Error bars represent S.E.M. **(B)** Selected frames of live-cell imaging of HeLa cells that were untreated (control), cells arrested with MSTNH2 alone, with MSTNH2+ LY294002, or with MSTNH2+ AZD5363, undergoing mitotic arrest and mitotic cell death. Green color denotes early-stage apoptotic cells. Arrowheads highlight individual cells for each sequence. Time denoted in hours: minutes. Bar, 50 µm. **(C)** Analysis of single-cell behaviors from time-lapse microscopy data in **(A)**. The duration of mitosis from mitotic entry to cytokinesis (controls) or cell death (MSTNH2-treated, ± PI3K pathway inhibitors) was measured for 55 individual cells per condition, with the mean time in mitosis listed for each condition. ****p < 0.0001.

Immunolabeling and Western blotting analyses suggested that PI3K pathway inhibition during mitotic arrest potentiated apoptosis greater than mitotic arrest alone (Figure 1; Supplementary Figure S1). To validate and extend the immunofluorescence and biochemical analysis data, we performed high-throughput long-term live-cell imaging using annexin V labeling and phase contrast microscopy (Figure 2). Quantification of cell death of HeLa cells revealed that while cells treated with LY294002 or AZD5363 alone had little effect on apoptosis (Figure 2A, blue and magenta lines, respectively), inhibition of PI3K or AKT in combination with MSTNH2-mediated mitotic arrest promoted and accelerated apoptosis over MSTNH2 alone (Figure 2A, red and orange lines, respectively). Tracking of individual cell fates over time as they entered and exited mitosis revealed that while untreated control HeLa cells required a little over an hour to successfully execute mitosis and cytokinesis (Figure 2B, panels a–e, arrowheads; Figure 2C), cells cultured in the presence of MSTNH2 remained arrested in mitosis for 15.7 h before displaying the characteristics typical of apoptosis (Figure 2B, panels f–j, arrowheads; Figure 2C). As reported by Gascoigne and Taylor (2008), Orth et al. (2008), Shi et al. (2008), there was a high degree of heterogeneity in the time that cells undergoing arrest were able to maintain mitotic arrest (Figure 2C). Measuring the duration of mitotic arrest from mitotic entry to apoptosis revealed that dual inhibition of PI3K/AKT signaling with AZD5363 or LY294002 dramatically reduced the duration of mitotic arrest relative to that with MSTNH2 alone (Figure 2B, panels k–t; Figure 2C). Most striking was the incidence of cells in the dual treatment cultures that lasted as little as 1 or 2 hours in mitosis before undergoing apoptosis (Figure 2C).

mTOR is a critical downstream effector of the PI3K/AKT pathway. To determine whether the protective effect of PI3K signaling acts through mTOR, cells were subjected to mitotic arrest in the presence or absence of the mTOR inhibitor rapamycin (Supplementary Figure S2). Rapamycin treatment, while maintaining pAKT levels, effectively abolished phosphorylation of the mTOR targets S6K and S6, confirming successful inhibition of mTOR activity, which was also observed with the MSTNH2+rapamycin combination (Supplementary Figures S2A,B). Time-resolved analyses of cell death revealed that while rapamycin alone had minimal effect on cell death, combination with MSTNH2 significantly enhanced apoptosis compared to that with either treatment alone (Supplementary Figure S2C). These results suggest that the PI3K pathway prevents precocious cell death during mitotic arrest partly through mTOR signaling.

### PI3K inhibition shifts the dynamics of cell death during mitotic arrest

**FIGURE 3:**
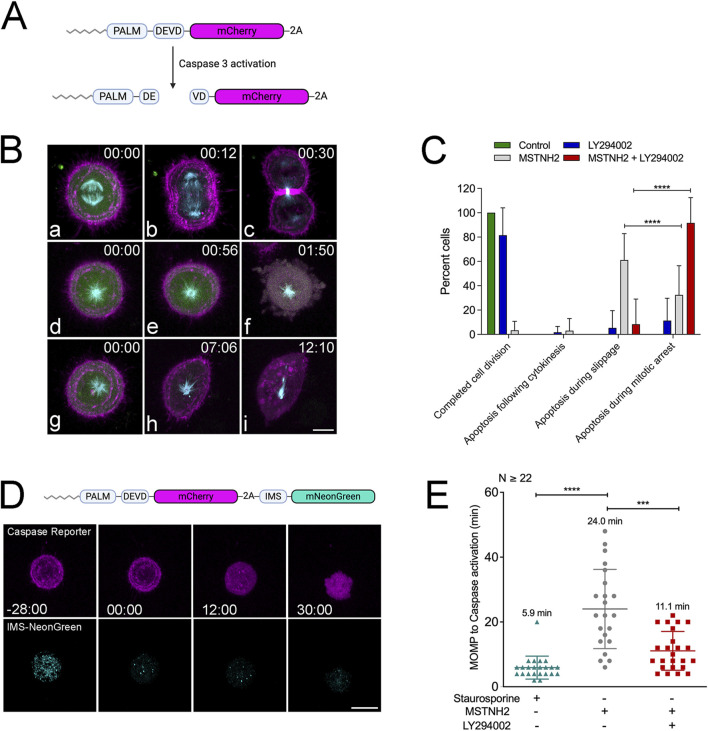
PI3K inhibition alters the kinetics of cell death in cells undergoing mitotic arrest. **(A)** Schematic of membrane-tagged caspase activity reporter. Caspase cleavage causes the red fluorescent protein to translocate from the membrane to the cytoplasm. **(B)** HeLa cells were co-transfected with caspase biosensor-2A-cerulean tubulin (red and cyan) and cyclin B-Venus (green), and then they were either imaged during normal mitotic progression or during mitotic arrest with MSTNH2 in the presence or absence of LY294002. **(a–c)** Normal cell division. Cyclin B was rapidly degraded at the metaphase–anaphase transition, followed by anaphase and cytokinesis. **(d–f)** Mitotic cell death, where the monaster remains symmetrical and cyclin levels are high when the caspase reporter is mobilized into the cytoplasm. **(g–i)** Cell death is associated with mitotic slippage, where cyclin B levels drop and the monopolar spindle assumes a polarized morphology. Time denoted in minutes: seconds, corresponding to frames in Supplementary Movies. Bar, 10 µm. **(C)** Quantification of cell fates. Results of >7 experimental replicates (≤5 cells per experiment) revealed that while the majority of arrested cells died by mitotic slippage followed by apoptosis, cells simultaneously treated with KSP and PI3K inhibitors died almost exclusively during mitotic arrest. Error bars represent SD. ****p < 0.0001. **(D)** HeLa cells imaged during mitotic arrest using a dual caspase/MOMP reporter, consisting of the membrane-targeted caspase reporter (magenta) and the mitochondrial-targeting domain of SMAC tagged with mNeonGreen fluorescent protein (IMS-mNeonGreen, cyan). IMS-mNeonGreen localizes to the mitochondrial intermembrane space until it is released into the cytoplasm during MOMP. Upon MOMP, the IMS-mNeonGreen marker rapidly transitions from a punctate mitochondrial localization to a faint cytoplasmic signal before caspase mobilization of the mCherry membrane marker. Time points denote the minutes: seconds prior to and following IMS-mNeonGreen translocation. Bar, 10 µm. **(E)** The time interval between MOMP and caspase activation was measured in HeLa cells expressing the two-color apoptosis biosensor and treated with either 2 μM staurosporine or arrested in mitosis in the absence or presence of PI3K inhibitors. Results of five experimental replicates (≥22 total cells); error bars represent SD. ***p = 0.0005 and ****p < 0.0001.

The results of morphological, biochemical, and long-term imaging analyses suggested that PI3K pathway inhibition accelerated the kinetics of apoptosis during mitotic delay (Figures 1, 2), and quantitation of the cell morphologies suggested that there was a loss of polarized cells (cells undergoing mitotic slippage) (Figure 1A). However, none of these approaches afforded the spatiotemporal resolution necessary to assess whether cells were undergoing cell death during mitotic arrest or during mitotic slippage. Therefore, a single-cell approach was employed to obtain high spatial and temporal resolution of the dynamics of mitotic arrest and cell death. A probe to simultaneously monitor apoptosis and cytoskeletal dynamics was generated, consisting of a caspase-3 cleavage site between an N-terminal myristoylation sequence and mCherry (Figure 3A) (Bhola and Simon, 2009) and Cerulean3-tubulin downstream of a peptide 2A sequence. Co-expressing this construct with cyclin B-Venus helped discriminate between cells dying during mitotic arrest and cells undergoing mitotic slippage. In cells undergoing normal divisions, cyclin B levels declined rapidly at the metaphase–anaphase transition and were undetectable as cells entered cytokinesis (Figure 3B, panels a–c; Supplementary Movie S1). Cells that died during mitotic arrest and treated with MSTNH2 were characterized by a strong cyclin signal and a radial array of dynamic microtubules at the time of caspase activation, as evidenced by the mobilization of the mCherry from the membrane to the cytoplasm (Figure 3B, panels d–f; Supplementary Movie S2). Cells that died during mitotic slippage were characterized by a loss of cyclin B signal and a polarized microtubule array before the mobilization of mCherry into the cytoplasm (Figure 3B, panels h–i; Supplementary Movie S3).

Quantification of cell fates in the absence or presence of KSP and PI3K inhibitors revealed that, consistent with the high-throughput imaging results (Figure 2), LY294002 alone did not affect mitotic progression (Figure 3C, blue bars). Treatment with MSTNH2 resulted in less than 4% of cells completing mitosis and cytokinesis, with greater than 60% of cells undergoing cell death during mitotic slippage and 33% during mitotic arrest (Figure 3C, gray bars). However, simultaneous treatment with MSTNH2 and LY294002 dramatically shifted the timing of cell death such that nearly 90% of cells died during mitotic arrest (Figure 3C, red bars). Thus, PI3K inhibition shifted the timing of cell death from apoptosis during mitotic exit to death during prometaphase arrest.

To track changes in the progression of apoptosis during mitotic arrest, the caspase reporter was co-expressed with a Mitochondrial Outer Membrane Permeabilization (MOMP) reporter consisting of the mitochondrial import sequence of SMAC fused to mNeonGreen (Figure 3D). IMS-mNeonGreen labels the mitochondria under normal conditions, but upon MOMP, the signal is rapidly dissipated into the cytoplasm just before caspase activation, allowing for a precise measurement of the timing between MOMP and caspase activation in living cells (Figure 3D; Supplementary Movie S4). When treated with a standard apoptotic inducer such as staurosporine, the MOMP: caspase activation interval is 5.9 min, at an SD ± 3.5. In contrast, in MSTNH2-treated cells, the gap between MOMP and caspase activation is dramatically longer (24 min) and more variable (SD ± 12.2, Figure 3E). However, there was a substantial reduction in the timing between IMS dispersion and caspase activation (11 min) in cells treated with both MSTNH2 and LY294002 (Figure 3E). Consistent with what was observed for the length of mitotic arrest (Figure 2C), the variation in response observed with MSTNH2 treatment alone (SD ± 12.2) was reduced when PI3K activity was also inhibited (SD ± 5.9, Figure 3E). Together, these data suggested that PI3K activity influenced both the kinetics and mechanics of cell death in cells experiencing mitotic delay.

**FIGURE 4:**
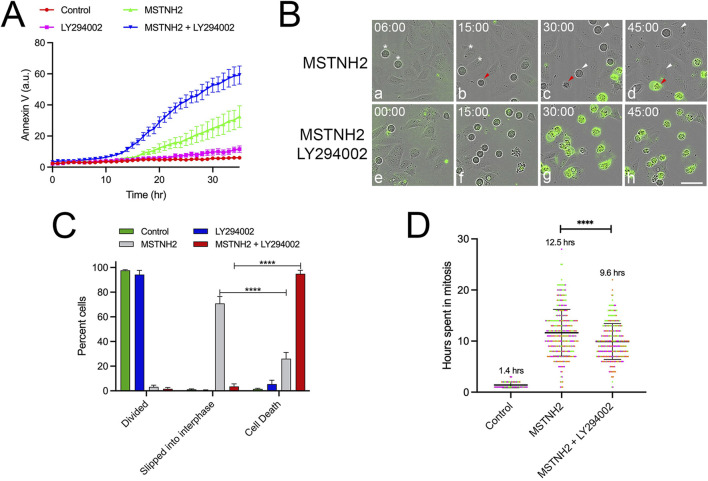
PI3K inhibition accelerates death in cells that survive mitotic arrest. **(A)** SiHa cells were cultured in the presence of fluorescent annexin V and combinations of KSP inhibitor (MSTNH2) and PI3K pathway inhibitors (LY294002). Apoptosis and cell morphology were monitored hourly by throughput microscopy (six wells per condition and three images captured per well). Disruption of PI3K signaling significantly accelerated apoptosis in SiHa cells undergoing mitotic arrest as compared to cell death in controls, MSTNH2-treated, or PI3K inhibition alone. Error bars represent S.E.M. **(B)** Selected frames of live-cell imaging of SiHa cells from the data in **(A)**. **(a–d)** SiHa cells arrested with MSTNH2 alone. White arrowheads denote cells that are arrested in mitosis only to slip back into mitosis, whereas the red arrowhead identifies a cell dying before slippage. In contrast, cells arrested with MSTNH2 in the presence of LY294002 **(e–h)** died during mitosis, as evidenced by both morphological changes and fluorescent annexin binding (green). Time denoted in hours: minutes. Bar, 50 µm. **(C)** Quantification of SiHa cell fates. Results of three experimental replicates (>100 cells per condition) revealed that while most arrested cells underwent slippage following arrest, cells simultaneously treated with both KSP and PI3K inhibitors died prior to slippage. Error bars represent SD. ****p < 0.0001. **(D)** Analysis of single-cell behaviors from time-lapse microscopy data in **(A,B)**. The duration of mitosis from mitotic entry to cytokinesis (controls) or cell death (MSTNH2-treated, ± LY294002) was measured for 55 individual cells per condition, with the mean time in mitosis listed for each condition. Duration of mitotic arrest from mitotic entry to cell death was measured for 60 individual cells per condition for three experimental replicates, with each experimental replicate represented by a different color. ****p < 0.0001.

To determine whether other cell lines were similarly sensitized by PI3K inhibition, the responses of three cervical cancer cell lines (C33A, Ca Ski, and SiHa), a sarcoma line (U-2 OS), and a non-transformed cell line (hTERT-RPE1) to mitotic arrest were assayed (Supplementary Figure S3). Tracking of individual cells (Supplementary Figure S3A) revealed differences both in the length of mitotic arrest and in cell fate (Supplementary Figure S3B). Compared to HeLa cells (which are highly sensitive to mitotic arrest), the majority of the hTERT-RPE1, U-2 OS, and SiHa cells survived mitotic arrest and slipped back into interphase (Supplementary Figure S3B). In terms of sensitivity to PI3K inhibition, while U-2 OS cells exhibited a mild potentiation in response to dual KSP/PI3K inhibition, C33A and hTERT-RPE1 cells exhibited no potentiation, and the Ca Ski cells were responsive to PI3K inhibition alone (Supplementary Figures S3C–F). However, SiHa cells exhibited a strong potentiation of cell death in response to dual KSP/PI3K inhibition (Figure 4A). Tracking of individual cell fates revealed that, in contrast to HeLa cells, 70% of the arrested cells survived mitotic delay and slipped to the interphase (Figures 4B,C). However, in the presence of the PI3K inhibitor, 95% of cells underwent apoptosis without slipping back into the interphase (Figures 4B,C) and spent less time arrested in mitosis before undergoing apoptosis (Figure 4D). Thus, even though HeLa and SiHa cells display different cell fate responses to mitotic arrest, both exhibited accelerated apoptosis in response to PI3K inhibition.

### Activated RAP1 alters cell fate in response to mitotic arrest

**FIGURE 5:**
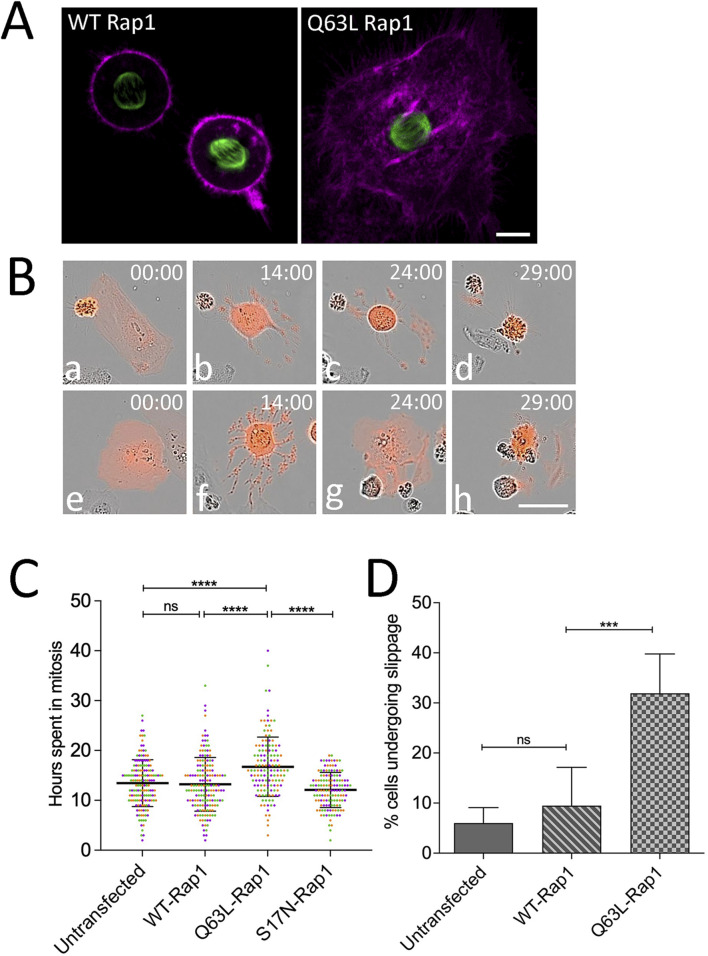
Activated Rap1 increases cell adhesion during mitosis and alters cell fate during mitotic arrest. **(A)** Confocal images of live HeLa cells co-expressing EGFP-tubulin (green), caspase reporter (magenta), and either WT or constitutively active Q63L Rap1. Notably, in contrast to cells expressing WT Rap1 (left), Q63L Rap1-expressing cells display a highly flattened morphology. Bar, 10 µm. **(B)** Analysis of single-cell behaviors from long-term time-lapse microscopy of HeLa cell responses to mitotic arrest in the presence of activated Rap1. Selected frames of live-cell imaging of HeLa cells expressing activated Rap1 caspase biosensor (red), cells treated with MSTNH2 and undergoing mitotic cell death **(a–d)**, and cell death following mitotic slippage and return to interphase **(e–h)**. Time denoted as hours: minutes. Bar, 50 µm. **(C)** Activated Rap1 increases the duration of mitotic arrest. HeLa cells expressing the apoptosis biosensor containing WT, constitutively active (Q63L), or dominant-negative (S17N) Rap1 were arrested in mitosis; cells were imaged every hour using an IncuCyte live-cell analysis system; and the interval from mitotic entry to caspase activation was measured. Results of three experimental replicates (∼50–60 cells per experiment, with each replicate represented by a different color) revealed a significant increase in the time frame from mitotic entry to caspase activation. Error bars represent SD. ****p < 0.0001. **(D)** Activated Rap1 significantly increased the percentage of cells slipping into the interphase as compared to WT. A total of 50–60 cells were scored per condition for three experimental replicates. Error bars represent SD. ***p = 0.0002.

Given that PI3K/AKT inhibition had profound effects on the cell fate during mitotic arrest in HeLa and SiHa cells (Figures 1–4), we sought to manipulate upstream modulators that may sustain this activity. The small GTPase Rap1 regulates diverse cellular processes, including cell adhesion and polarity, and has also been implicated in PI3K pathway activation by recruiting the p110 catalytic subunit to the membrane and enhancing PIP3-mediated AKT phosphorylation (Dao et al., 2009; Kortholt et al., 2006). To better understand how PI3K activity may be maintained or regulated during mitotic delay, wild-type or constitutively active Rap1 (Q63L) was co-expressed with the caspase reporter and EGFP-tubulin in HeLa cells and imaged live by confocal microscopy. While cells expressing WT Rap1 underwent characteristic rounding during mitosis (Figure 5A, left), cells expressing Q63L-Rap1 remained flattened during mitosis (Figure 5A, right), which is consistent with previous reports suggesting that the flat cell morphology was induced by overexpression of Rap1 (Dao et al., 2009; Lancaster et al., 2013). However, in contrast to earlier reports that Rap1-mediated flattening resulted in errors in cytokinesis (Dao et al., 2009), in our study, more than 90% of cells expressing Q63L Rap1 underwent successful division. Long-term monitoring of individual cells co-expressing the caspase reporter and either WT or Q63L Rap1 (Figure 5B) revealed that cells expressing Q63L-Rap1 persisted in mitosis longer than those expressing WT-Rap1 (Figure 5C) and that several cells expressing Q63L Rap1 completely slipped back to interphase after prolonged mitotic arrest (Figure 5B, panels f and g, and D), where they underwent cell death within 17 h (Figure 5B, panels e–h, and C). Expression of a dominant-negative mutant of Rap1 (S17N) did not affect the ability of cells to sustain mitotic arrest (Figure 5C) as Rap1 activity is normally downregulated during mitosis (Dao et al., 2009). Thus, inhibition of endogenous Rap1 activation had no effect on cells undergoing mitotic arrest.

**FIGURE 6:**
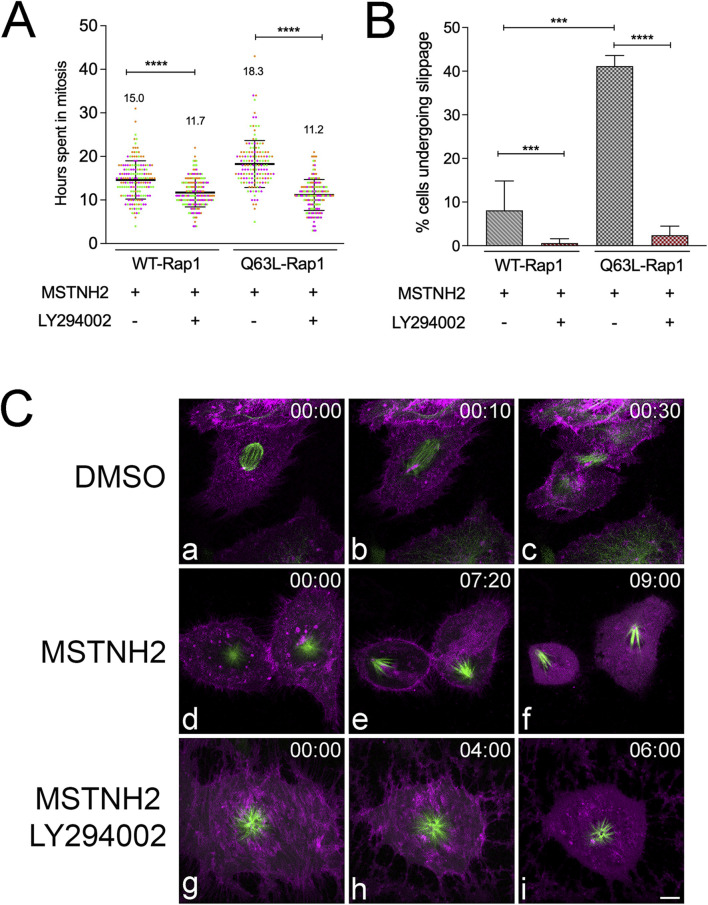
Activated Rap1 promotes persistence and survival of mitotic arrest through PI3K. **(A)** HeLa cells expressing the apoptosis biosensor containing either WT or Q63L Rap1 were arrested in mitosis in the absence or presence of PI3K inhibitors. Cells were imaged every hour using an IncuCyte live-cell analysis system, and the interval from mitotic entry to caspase activation was measured. Results of three experimental replicates (∼50–60 cells per experiment, with each replicate represented by a different color) revealed a significant decrease in the time frame of mitotic entry to caspase activation when treated with the PI3K inhibitor. The mean time in mitosis is listed for each condition. Error bars represent SD. ****p < 0.0001. **(B)** PI3K inhibition significantly decreased the percentage of Q63L Rap1-expressing cells that slipped into interphase. A total of 50–60 cells were scored per condition for three experimental replicates. Error bars represent SD. ***p = 0.0008 and ****p < 0.0001. **(C)** Cell fates of HeLa cells expressing Q63L Rap1, the caspase biosensor (magenta), and EGFP-tubulin (green). **(a–c)** Cell division in the absence of mitotic arrest. **(d–f)** Mitotic slippage and cell death, with cells exhibiting characteristic polarized monopolar spindles **(e)** prior to mobilization of the caspase reporter into the cytoplasm **(f)**. **(g–i)** Cell death during mitotic arrest, where the mCherry caspase reporter is released into the cytoplasm, with the spindle still displaying a characteristic monastrol morphology and mitotic microtubule dynamics **(i)**. Time denoted in hours: minutes. Bar, 10 µm.

To determine whether the protective effects of activated Rap1 were PI3K-dependent, HeLa cells expressing WT or Q63L Rap1 were treated with MSTNH2 ± LY294002. As shown in Figure 6A, PI3K inhibition reversed the protective effects of activated Rap1 as cells were surviving only ∼11 ± 3.6 h after entering mitosis when PI3K activity was inhibited, which is in contrast to 18 ± 5.4 h when LY294002 was absent. Similarly, the percentage of Q63L Rap1 cells re-entering the interphase decreased significantly in the absence of PI3K (Figure 6B). Live-cell confocal imaging also confirmed that while cells expressing Q63L-Rap1 were able to complete cell division (Figure 6C, panels a–c; Supplementary Movie S5), cells arrested in mitosis with MSTNH2 that did not slip back into the interphase died during mitotic slippage, as evidenced by the change in microtubule dynamics and spindle polarization (Figure 6C, panels d–f; Supplementary Movie S6). In contrast, in the presence of a PI3K inhibitor, Q63L Rap1-expressing cells that underwent arrest with MSTNH2 died during prometaphase arrest (Figure 6C, panels g–i; Supplementary Movie S7). Together, these findings confirmed our speculation that activated Rap1 modulates cell survival during mitotic arrest in a PI3K-dependent manner.

To determine whether Rap1’s PI3K-dependent effects on cell fate might be mediated through its known role in regulating cell adhesion (Bos et al., 2001; Reedquist et al., 2000; Shah et al., 2019), we inhibited focal adhesion kinase (FAK) activity in cells arrested in mitosis. HeLa cells were treated with FAK inhibitor defactinib (FAKi) alone or in combination with MSTNH2, and the kinetics of cell death were quantified by long-term microscopy. Similar to what was observed for PI3K, combined inhibition of FAK and KSP promoted cell death to a degree greater than that when blocking each individually (Supplementary Figure S4A). However, FAK inhibition had no significant effect on the length of mitotic arrest in Q63L Rap1-expressing cells (Supplementary Figure S4B) or the percentage of cells that were able to completely exit to the interphase before undergoing apoptosis (Supplementary Figure S4C). We conclude that Rap1’s effects on cell fates in response to mitotic arrest were independent of FAK and likely worked independently to stimulate cell adhesion and PI3K activity.

## Discussion

Antimitotic chemotherapeutics such as taxanes and vinca drugs have been used in the clinic for 50 years, but despite their efficacy, their use is associated with side effects on both dividing and non-dividing cells and development of resistance (Monzó et al., 1999; Rowinsky et al., 1993a; Rowinsky et al., 1993b). For these reasons, KSP represents a favorable antimitotic target to block cancer cell proliferation since KSP operates only during mitosis and, thus, does not have the same effects on terminally differentiated cells (Shahin and Aljamal, 2022). Despite their promise as an alternative to taxanes and the near-universal requirement of class-5 kinesins for spindle assembly, KSP inhibitors have not proven to be more effective than traditional taxanes in clinical trials (Jones et al., 2013; Mitchison, 2012). The goal of this study was to determine whether a combinatorial approach could increase the efficacy of KSP inhibitors by targeting a second pathway that is itself not essential. Results of morphological, biochemical, and live-cell analyses revealed that simultaneous inhibition of spindle bipolarity and the PI3K signaling pathway greatly accelerated both the timing of apoptosis in mitotically arrested HeLa cells and the kinetics of the intrinsic apoptotic pathway. Thus, these findings suggest that the efficacy of KSP inhibitors may be significantly improved when used in combination with other drugs and that the wide heterogeneity of cellular responses observed both *in vitro* and *in vivo* may be a function of cellular signaling pathways that promote cell survival and metabolism.

Combinatorial chemotherapeutic approaches to treating cancer are not new, and indeed, PI3K is a potential target in many studies (West et al., 2002) including antimitotic drugs (Hu et al., 2002; Wallin et al., 2012). Biochemical and morphological analyses confirmed that treatment of HeLa cells with a novel KSP inhibitor in combination with PI3K/AKT/mTOR inhibitors resulted in potentiated cell death over the treatment of these small molecules individually (Figure 1; Supplementary Figures S1, S2), supporting earlier results that PI3K inhibitors sensitize cancer cells to antimitotic drugs (Hou et al., 2012). Indeed, activating mutations in p110α confers increased resistance to paclitaxel (Isakoff et al., 2005), and upregulation of AKT activity increases resistance to microtubule inhibitors (VanderWeele et al., 2004). However, these studies did not lend any mechanistic insights as to why PI3K inhibition increases cell death during mitotic arrest. High-throughput quantitative microscopy confirmed these findings with finer time resolution (Figure 2A) and helped measure the ability of single cells to sustain mitotic arrest in the presence or absence of PI3K/AKT activity (Figures 2B,C). Notably, tracking of the fates of individual cells demonstrated that in the absence of PI3K activity, cells were unable to sustain mitotic arrest and died sooner after entering mitosis compared to cells treated with MSTNH2 alone (Figures 2B,C). Thus, the increased rate of cell death observed in the combinatorial treatments was directly associated to cells in mitosis and their ability to sustain mitotic arrest.

**FIGURE 7:**
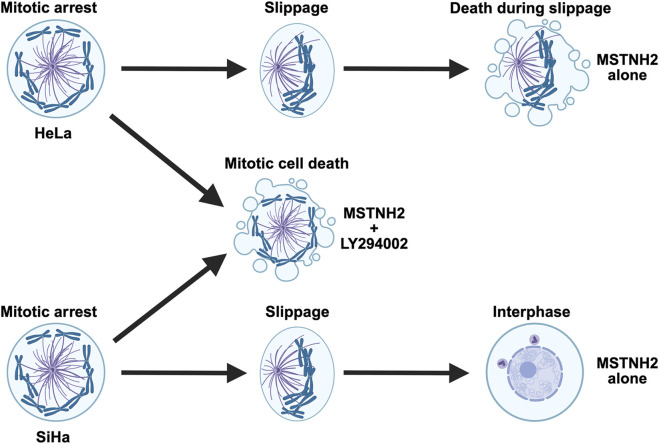
Summary of cell fates in response to mitotic arrest for PI3K-sensitive cell lines. HeLa cells arrested in mitosis with a monopolar spindle will eventually begin to slip out of mitosis as the cyclin B levels drop below a threshold level. The mitotic spindle polarizes as microtubule dynamics shift toward interphase levels, and it is during slippage that HeLa cells undergo apoptosis. In the absence of PI3K activity, HeLa cells undergo apoptosis while still in mitotic arrest. In contrast, SiHa cells survive both mitotic arrest and slippage and return to the interphase as a tetraploid cell. However, in the absence of PI3K activity, SiHa cells fail to maintain mitotic arrest and die before slippage.

HeLa cells die during mitotic slippage (Orth et al., 2008), and analyses of populations of fixed and immunolabeled cells suggest that when PI3K signaling is inhibited, there is a notable loss of cells undergoing mitotic slippage (Figure 1A). However, to better resolve cell death at both spatial and temporal scales, fluorescent reporters for caspase activity, mitotic spindle, and M-phase were co-expressed, and the fates of single cells were quantified (Figure 3). Combined treatment revealed a dramatic shift in cell fates, with HeLa cells dying during prometaphase arrest in the absence of PI3K activity, as opposed to dying during mitotic slippage (Figure 3C; Figure 7). PI3K/AKT inhibition also altered the kinetics by which cells undergoing mitotic arrest progressed through apoptosis. Measurement of the timing between MOMP and caspase activation revealed that, in contrast to cells treated with staurosporine, cells undergoing mitotic arrest have a significantly longer gap between MOMP and caspase activation, with a wide degree of variation at the individual cell level (Figure 3E). However, PI3K inhibition dramatically decreased the timing between MOMP and caspase activation such that it was not significantly different from that with staurosporine treatment (Figure 3E). While the change in the kinetics of apoptosis does not account for the overall differences in the timing of mitotic arrest between cells possessing or lacking PI3K activity (Figure 2C), it does implicate PI3K in modulating the intrinsic apoptotic pathway during mitotic delay. Together, these results suggest that blocking PI3K activity resulted in quantitative and qualitative changes in the way HeLa cells undergo apoptosis in response to prolonged mitotic delay.

While there is little variation in the action of microtubule and KSP inhibitors on cells, there is substantial heterogeneity in responses across cell lines along with variation at the individual cell level, both *in vitro* and *in vivo* (Gascoigne and Taylor, 2008; Orth et al., 2011; Orth et al., 2008; Shi et al., 2008). Examination of other cervical cancer cell lines, an osteosarcoma line, and a non-transformed cell line confirmed this heterogeneity, in their response to mitotic arrest (Supplementary Figures S3A,B) and in terms of their sensitivity to simultaneous PI3K inhibition (Figure 4; Supplementary Figures S3C–F). Of the cells tested, SiHa cells exhibited sensitivity to PI3K inhibition during mitotic delay that mirrored that of HeLa cells (Figure 4). SiHa cells normally survive mitotic arrest, slipping completely back into the interphase (Figures 4B,E). However, PI3K inhibition shifted the fates of cells undergoing mitotic arrest such that SiHa cells died in mitosis (Figures 4B,C; Figure 7). Whether or not cell lines were sensitized to PI3K was not correlated with p53 status or the presence or absence of mutations in PI3K or PTEN (Supplementary Figure S3B). Thus, while there are likely other commonalities shared by HeLa and SiHa cells that render them sensitive to PI3K inhibition during mitotic arrest, PI3K activity alone is not the primary determinant of cell fate responses to mitotic delay.

The notion that there may be a link between cell adhesion, PI3K, and persistence during mitotic delay is supported by studies demonstrating that integrin signaling via PI3K could protect cells against paclitaxel and vincristine (Aoudjit and Vuori, 2001; Westhoff and Fulda, 2009) and that activated mutants of PI3K protected breast cancer cells against anoikis (Isakoff et al., 2005; Schmidt et al., 2002). Rap1 has previously been identified as a modulator of PI3K, either exerting positive or negative regulation depending on different cell types and different stimuli (Bos et al., 2001); Rap1 interacts with class-I PI3Ks, promoting PIP3 synthesis and activating AKT (Kortholt et al., 2010; Shah et al., 2019). In HeLa cells, constitutively active Rap1 not only increased the timing from mitotic entry to cell death, but also increased the percentage of cells that slipped back into the interphase before cell death (Figures 5B–D, 6). Moreover, this effect was dependent on PI3K (Figure 6), suggesting that Rap1 promotes PI3K activity in HeLa cells. Cross-talk between Rap1 and FAK during integrin-mediated cell adhesion has been demonstrated (Chang et al., 2019; Su and Wu, 2021), and while FAK inhibition showed a dramatic increase in cell death during mitotic arrest, there were no differences in cell fates in the presence of activated Rap1 (Supplementary Figure S4). It suggests that Rap1-mediated cell spreading and protection from mitotic cell death are not dependent on FAK but might instead be due to alternative pathways through which Rap1 promotes adhesion and PI3K activity (Ross et al., 2012).

## Conclusion

The results of these lines of experimentation suggest that for a subset of cancers, inhibition of PI3K/AKT/mTOR signaling can dramatically increase the efficacy of kinesin-5 inhibitors in driving mitotic cell death. Acceleration of apoptosis occurred in cell types that normally survived extended mitotic delay and in those that typically died as they underwent mitotic slippage (Figure 7). In addition to reducing the time that cells could maintain mitotic arrest, PI3K inhibition affected the kinetics of mitochondrial-based cell death, dramatically compressing the lag time between MOMP and caspase activation. Together, these results indicate a mechanism by which anti-apoptotic signaling by PI3K helps determine cellular responses to antimitotic drugs.

## Data Availability

The raw data supporting the conclusions of this article will be made available by the authors, without undue reservation.

## Supplementary material

SUPPLEMENTARY MOVIE S1 Mitosis and cytokinesis in a control HeLa cell. Time-lapse confocal movie of a HeLa cell expressing cyclin B-Venus (green), membrane-tagged mCherry caspase reporter (magenta), and cerulean3-tubulin (cyan) in the presence of 0.1% DMSO. Four image planes were acquired at 1-μm intervals every 2 min and played back at 10 frames/second.

SUPPLEMENTARY MOVIE S2 Mitotic cell death in a HeLa cell. Time-lapse confocal movie of a HeLa cell expressing cyclin B-Venus (green), membrane-tagged mCherry caspase reporter (magenta), and cerulean3-tubulin (cyan) and arrested in mitosis with 500 nM MSTNH2. Four image planes were acquired at 1-μm intervals every 2 min and played back at 10 frames/second.

SUPPLEMENTARY MOVIE S3 Cell death during mitotic slippage in a HeLa cell. Time-lapse confocal movie of a HeLa cell expressing cyclin B-Venus (green), membrane-tagged mCherry caspase reporter (magenta), and cerulean3-tubulin (cyan) and arrested in mitosis with 500 nM MSTNH2. Four image planes were acquired at 1-μm intervals every 2 min and played back at 10 frames/second.

SUPPLEMENTARY MOVIE S4 Simultaneous imaging of MOMP and caspase activation during mitotic cell death. Time-lapse confocal movie of a HeLa cell expressing membrane-tagged mCherry caspase reporter (Magenta) and IMS-NeonGreen (cyan) and arrested in mitosis with 500 nM MSTNH2. Six image planes were acquired at 1-μm intervals every 2 min and played back at 10 frames/second.

SUPPLEMENTARY MOVIE S5 Mitosis and cytokinesis in a HeLa cell expressing Q63L Rap1. Time-lapse confocal movie of a HeLa cell expressing Q63L Rap1, membrane-tagged mCherry caspase reporter (magenta), and EGFP-tubulin (green) progressing through mitosis and cytokinesis in the presence of 0.1% DMSO. Four image planes were acquired at 1-μm intervals every 2 min and played back at 10 frames/second.

SUPPLEMENTARY MOVIE S6 Cell death during mitotic slippage in HeLa cells expressing Q63L Rap1. Time-lapse confocal movie of two HeLa cells expressing Q63L Rap1, membrane-tagged mCherry caspase reporter (magenta), and EGFP-tubulin (green) and arrested in mitosis with 500 nM MSTNH2. After transfection, cells were treated with 500 nM MSTNH2 to induce mitotic arrest. Four image planes were acquired at 1-μm intervals every 2 min and played back at 10 frames/second.

SUPPLEMENTARY MOVIE S7 Mitotic cell death in a HeLa expressing Q63L Rap1 in the presence of a PI3K inhibitor. Time-lapse confocal movie of a HeLa cell expressing Q63L Rap1, membrane-tagged mCherry caspase reporter (magenta), and EGFP-tubulin (green). After transfection, cells were treated with 500 nM MSTNH2 to induce mitotic arrest in the presence of 20 μM LY294002. Four image planes were acquired at 1-μm intervals every 2 min and played back at 10 frames/second.
