# Supplementary material for: Factors that determine cell fate in mitotically arrested cancer cells
Source: Front Cell Dev Biol. 2026 Jan 12;13:1691574. doi: 10.3389/fcell.2025.1691574 (PMC12832742; doi:10.3389/fcell.2025.1691574)
Supplement: Supplementary file 3 [file DataSheet1.pdf]

Supplementary Material

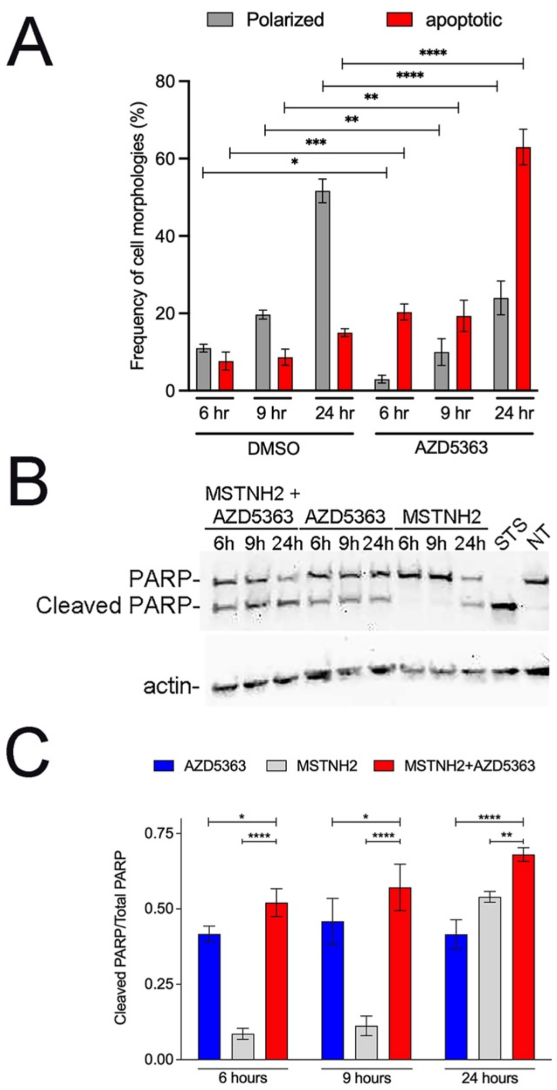

Supplementary Figure S1. Disruption of AKT signaling promotes mitotic cell death. (A) HeLa cells were treated with 500 nM MSTNH2 in the absence or presence of 20 µM AZD5356, and at the denoted time points, cells were fixed and probed for morphological markers for mitotic arrest and apoptosis as shown in Figure 1A. Graph depicts a representative experiment with ~200 cells scored per replicate (three replicates per condition per experiment). Error bars represent SD. **(B)** HeLa cell lysates were generated from cultures treated with 500 nM MSTNH2, 20 µM AZD5356, MSTNH2+AZD5356 or 1 µM staurosporine and probed for PARP and probed for cleaved PARP and actin. **(C)** Quantification of PARP conversion from its full-length (116 kd) to the cleaved 89kd proteolyzed product; error bars represent SD of three independent experiments. ** $p<0.01$, **** $p<0.0001$.

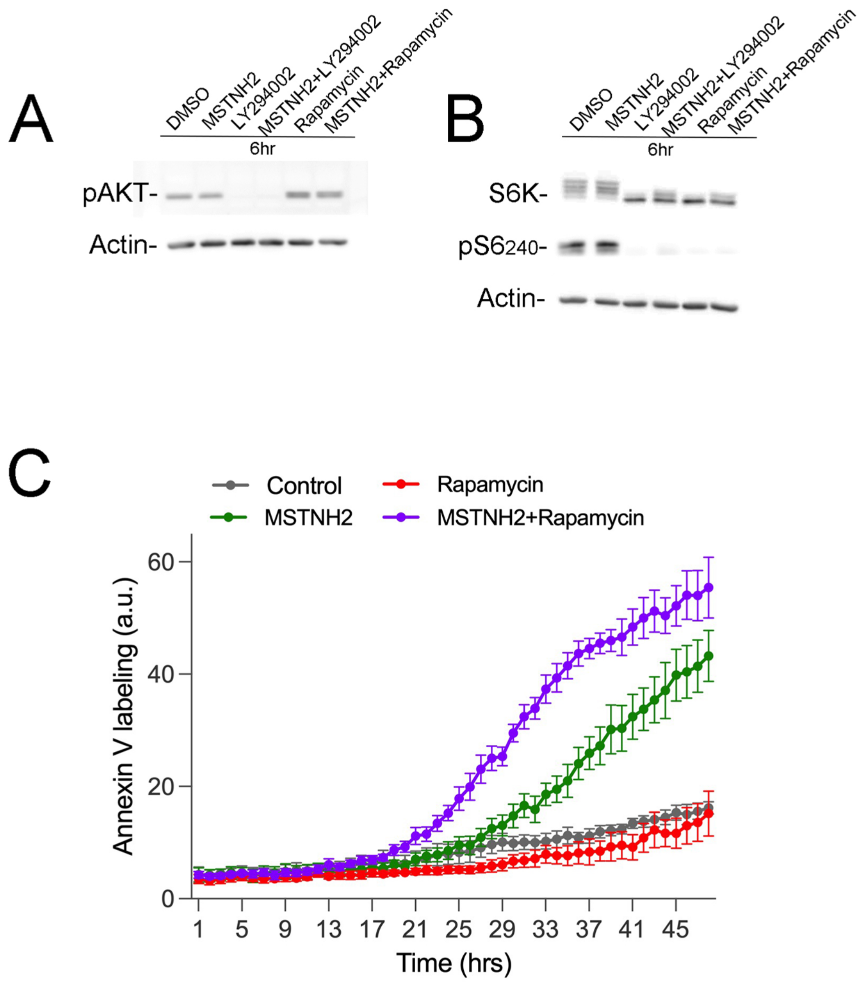

Supplementary Figure S2. **Disruption of mTOR signaling promotes mitotic cell death (A)** Western blot of HeLa cells lysates treated with 500 nM MSTNH2, 500 nM Rapamycin, 20 µM LY294002 or combinations thereof for up to 6 hr and probed for phosphorylated AKT (pAKT) and actin loading control. **(B)** Western blot analysis of downstream mTORC1 signaling. **(C)** HeLa cells were cultured in the presence of fluorescent Annexin V and combinations of KSP inhibitor (MSTNH2) and mTOR inhibitor (Rapamycin). Apoptosis was monitored hourly by throughput microscopy (4 wells per condition, 3 data points per well). Disruption of mTOR significantly accelerated apoptosis in mitotically arrested HeLa cells as compared to cell death in control, and MSTNH2 or Rapamysin treatment alone. Error bars represent S.E.M.

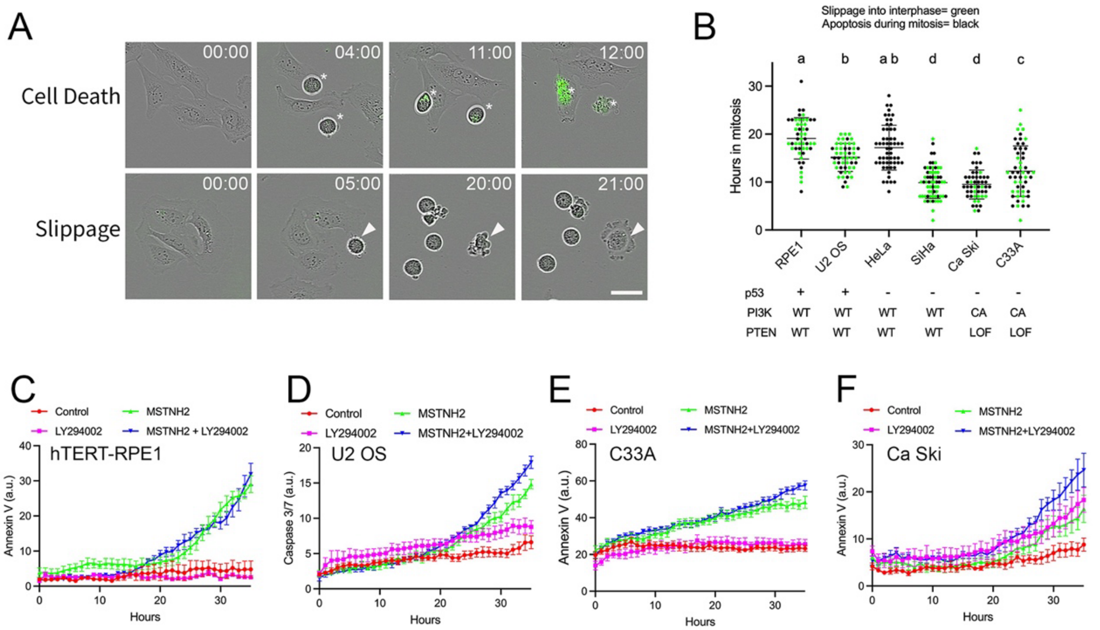

Supplementary Figure S3. Cell death responses to PI3K inhibition in different cell types. (**A and B)** Individual cells were tracked to measure determine the cell fates of cells arrested in mitosis with MSTNH2 as well as the time from mitotic entry to cell death or slippage, measured for ≥50 individual cells per condition. Cell fates are denoted as black for cell death and green for mitotic slippage and return to interphase. Significance determined by one-way ANOVA with Tukey–Kramer post hoc test, significant differences between samples (≤ 0.05) denoted with different case letters. (**C-F**) hTERT-RPE1, U2 OS, C33A and Ca Ski cells were cultured in 0.1% DMSO or combinations of KSP (MSTNH2) and PI3K (LY294002) inhibitors and monitored hourly by throughput microscopy (6 wells per condition, 3 images captured per well) for apoptosis using either fluorescent Annexin V (**C, E-F**) or a Caspase 3/7 reporter (**D**). Error bars represent S.E.M. In contrast to HeLa cells (Figure 2) or SiHa cells (Figure 4), these cell lines did not exhibit enhanced or accelerated cell death when subjected to both mitotic arrest and PI3K inhibition.

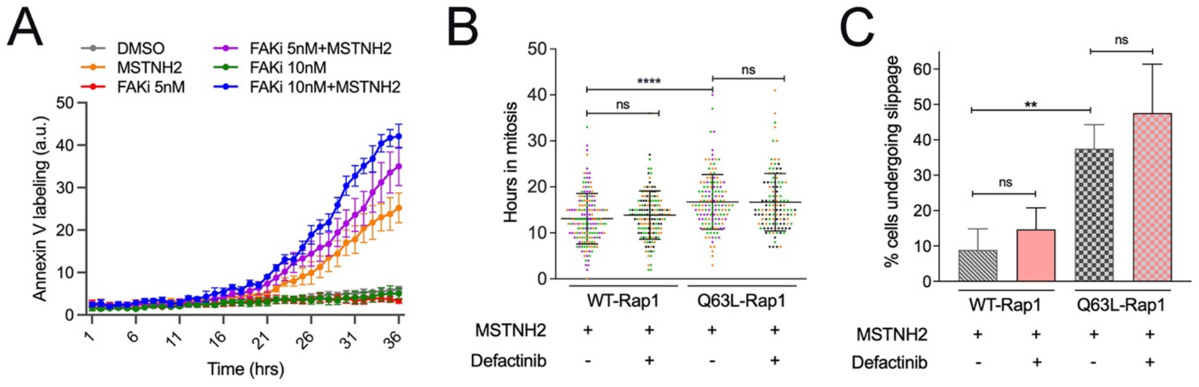

Supplementary Figure S4. Activated Rap1 promotes cell survival independently of FAK. (A) Cell death kinetics in HeLa cells in the absence or presence of a Focal Adhesion Kinase inhibitor. HeLa cells were cultured in the presence of fluorescent Annexin V and combinations of MSTNH2 and 5 nM or 10 nM FAK inhibitor (Defectinib). Apoptosis was monitored hourly using an IncuCyte live cell analysis system (3 wells per condition, 9 data points per well). Disruption of FAK accelerated apoptosis in mitotically arrested HeLa cells in dose-dependent manner as compared to cell death in control, and mitotic arrest or FAK inhibition alone. **(B)** HeLa cells co-expressing the Caspase reporter and either WT or Q63L Rap1 were arrested in mitosis in absence or presence of FAK inhibitor. Cells were imaged every hour using an IncuCyte live cell analysis system, and the interval from mitotic entry to caspase activation was measured. Results of three-experimental replicates (~50-60 cells per experiment, each replicate represented by a different color) revealed no significant difference in the time frame of mitotic entry to caspase activation when treated with FAK inhibitor. Error bars represent SD. **** p<0.0001. **(C)** FAK inhibition had no significant difference in the percentage of cells slipping into interphase. 50-60 cells were scored per condition for three experimental replicates; error bars represent SD. ** p<0.01.
